# AepG is a glucuronosyltransferase involved in acidic exopolysaccharide synthesis and contributes to environmental adaptation of *Haloarcula hispanica*

**DOI:** 10.1016/j.jbc.2023.102911

**Published:** 2023-01-13

**Authors:** Caixia Pei, Hua Lu, Jiayin Ma, Jerry Eichler, Ziqiang Guan, Linlu Gao, Li Liu, Hui Zhou, Jinghua Yang, Cheng Jin

**Affiliations:** 1 State Key Laboratory of Mycology, Institute of Microbiology, Chinese Academy of Sciences, Beijing, China; 2 University of Chinese Academy of Sciences, Beijing, China; 3 Department of Pharmacology, UT Southwestern Medical Center, Dallas, Texas, USA; 4 Glycomics and Glycan Bioengineering Research Center (GGBRC), College of Food Science and Technology, Nanjing Agricultural University, Nanjing, China; 5 Department of Life Sciences, Ben Gurion University of the Negev, Beersheva, Israel; 6 Department of Biochemistry, Duke University Medical Center, Durham, North Carolina, USA

**Keywords:** archaea, carbohydrate biosynthesis, dolichol phosphate, enzyme, glucuronic acid, glycosyltransferase, *Haloarcula hispanica*, ACN, acetonitrile, aepG, archaeal exopolysaccharide glucuronosyltransferase, CBD, cellulose-binding domain, DolP, dolichol phosphate, GlcA, glucuronic acid, OPRAL, One-Pot Release and Labeling, PAS, Periodic Acid-Schiff’s, PMP, 1-phenyl-3-methyl-5-pyrazolone

## Abstract

The attachment of a sugar to a hydrophobic lipid carrier is the first step in the biosynthesis of many glycoconjugates. In the halophilic archaeon *Haloarcula hispanica*, HAH_1206, renamed AepG, is a predicted glycosyltransferase belonging to the CAZy Group 2 family that shares a conserved amino acid sequence with dolichol phosphate mannose synthases. In this study, the function of AepG was investigated by genetic and biochemical approaches. We found that *aepG* deletion led to the disappearance of dolichol phosphate-glucuronic acid. Our biochemical assays revealed that recombinant cellulose-binding, domain-tagged AepG could catalyze the formation of dolichol phosphate-glucuronic acid in time- and dose-dependent manners. Based on the *in vivo* and *in vitro* analyses, AepG was confirmed to be a dolichol phosphate glucuronosyltransferase involved in the synthesis of the acidic exopolysaccharide produced by *H. hispanica*. Furthermore, lack of *aepG* resulted in hindered growth and cell aggregation in high salt medium, indicating that AepG is vital for the adaptation of *H. hispanica* to a high salt environment. In conclusion, AepG is the first dolichol phosphate glucuronosyltransferase identified in any of the three domains of life and, moreover, offers a starting point for further investigation into the diverse pathways used for extracellular polysaccharide biosynthesis in archaea.

Microbial exopolysaccharides (EPSs) are extracellular carbohydrate polymers involved in cell growth, adherence, nutrient storage, and barrier protection and biofilm formation, all of which are important for microorganism adaptation and survival (1, 2, 3, 4, 5, 6, 7, 8). EPSs produced by bacteria have been extensively studied, having economic importance as emulsifying, viscosifying, and chelating agents, and as anti-oxidants (9, 10, 11, 12, 13). Like bacteria, haloarchaea belonging to the genera *Haloferax*, *Halococcus*, *Haloarcula*, *Natronococcus*, *Haloterrigena*, and *Halobacterium* also produce EPSs (14, 15, 16, 17, 18, 19). However, little is known about the genes or pathways that these archaea use for EPS biosynthesis.

*Haloarcula hispanica* is an extremely halophilic archaeon originally isolated from a solar saltern in Spain (20). Previously, we showed that *H. hispanica* can produce an acidic EPS that protects the cells against harsh environments (21). Although two glycosyltransferase-encoding genes (*HAH_1662* and *HAH_1667*) involved in the synthesis of the acidic EPS were identified (21), the complete pathway used for the synthesis of this EPS remains to be delineated.

Analysis of the *H. hispanica* genome revealed that the *HAH_1202*-*HAH_1214* cluster contains several genes encoding putative glycosyltransferases, including *HAH_1206* (22). In this report, the function of the *HAH_1206* was investigated. Our results revealed that the protein encoded by *HAH_1206* is a dolichol phosphate (DolP) glucuronosyltransferase needed for the incorporation of glucuronic acid (GlcA) and sulfated-GlcA into the acidic EPS. We also found that *HAH_1206* was required for the adaptation of *H. hispanica* to high salt. As the first DolP glucuronosyltransferase shown to be involved in EPS synthesis, *HAH_1206* and its protein product were renamed *aepG* (archaeal exopolysaccharide glucuronosyltransferase) and AepG, respectively.

## Results

### *H. hispanica* HAH_1206 encodes a putative glycosyltransferase

**Figure 1:**
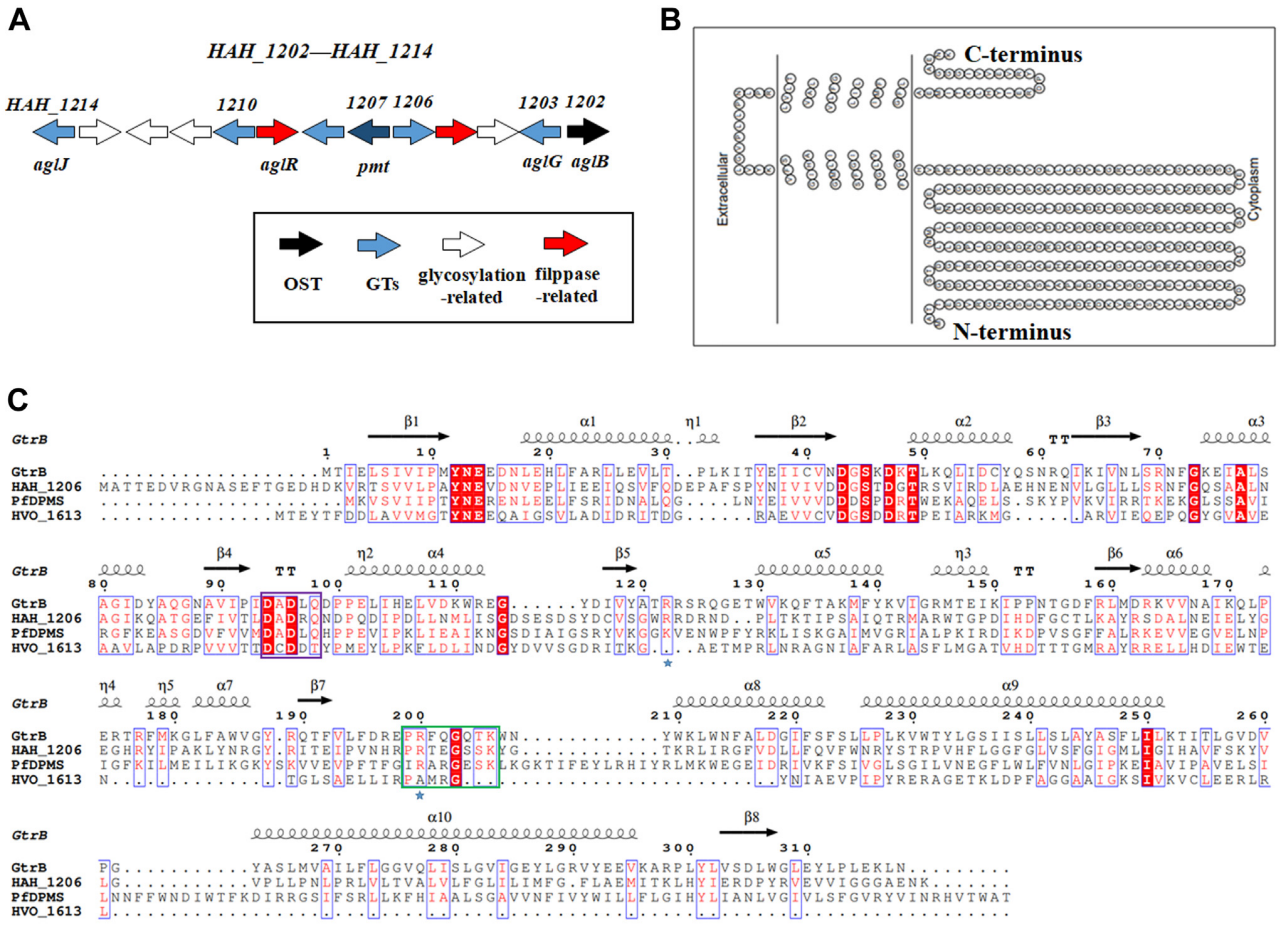
**Bioinformatical analysis of *Haloarcula hispanica* HAH_1206.***A*, schematic depiction of the *H. hispanica* genomic regions near *HAH_1206*. *B*, topology of HAH_1206 predicted by Topo2 software at the SOSUI server (http://www.sacs.ucsf.edu/TOPO2/). *C*, alignment of *H. hispanica* HAH_1206 with *Synechocystis* GtrB, *Pyrococcus furiosus* PF0058, and *Haloferax. volcanii* HVO_1613. The conserved DADXQX motif of family 2 inverting glycosyltransferases is highlighted by a *purple box*. The acceptor-loop of *P. furiosus* PF0058 is highlighted by a *green box*. Conserved residues required for the binding of lipid-phosphate acceptors are marked with a *blue asterisk*.

Homology searches and conserved domain analysis identified four gene clusters encoding putative glycosyltransferases potentially involved in protein glycosylation and/or EPS synthesis in *H. hispanica*, including *HAH_1202*-*HAH_1214*, *HAH_0486*-*HAH_0496*, *HAH_1567*-*HAH_1573*, and *HAH_1661*-*HAH_1667* (21, 22). Among these, the cluster spanning from *HAH_1202* to *HAH_1214* (Fig. 1*A*) includes a gene (*HAH_1202*) encoding a homolog of the archaeal oligosaccharyltransferase and genes encoding homologs of proteins involved in N-glycosylation in *Haloferax volcanii*, another haloarchaeon where the pathway of N-glycosylation has been delineated (23, 24, 25, 26). This *H. hispanica* gene cluster also includes *HAH_1206*, which encodes a putative protein (HAH_1206) previously annotated as a CAZy (carbohydrate-active enzyme) Group 2 glycosyltransferase (GT-2) family protein (27, 28). The TOPO2 Transmembrane Protein Display algorithm (http://www.sacs.ucsf.edu/cgi-bin/open-topo2.py/) predicted that HAH_1206 is a membrane protein containing two transmembrane domains at the C-terminal end and a cytoplasmically oriented N-terminal catalytic domain (Fig. 1*B*). 3D modeling prediction (https://swissmodel.expasy.org/interactive) revealed that HAH_1206 is similar to *Synechocystis* GtrB, a GT-2 family protein that catalyzes the transfer of glucose from UDP-glucose to undecaprenylphosphate and to *Pyrococcus furiosus* DolP mannose synthase PF0058, both of which have solved structures (Fig. 1*C*) (29, 30). These similarities include the DADXQX signature motif, the acceptor loop, and the active site of family 2 inverting glycosyltransferases (31). Additionally, HAH_1206 is also similar to *H. volcanii* HVO_1613, a DolP glycosyltransferase not involved in N-glycosylation (32). Given all these enzymes are glycosyltransferases, catalyzing the transfer of the sugar from a nucleoside-charged diphospho-sugar donor to a phosphorylated polyprenol lipid carrier, it is likely that HAH_1206 is also a glycosyltransferase involved in the production of a lipid-linked sugar (Fig. 1*C*).

### Deletion of *H. hispanica* HAH_1206

Quantitative RT-PCR analysis revealed that *HAH_1206* was transcribed during log phase and that its level of expression was 78% of that of the 16S RNA gene, suggesting that *HAH_1206* is expressed in *H. hispanica*. To investigate the function of *HAH_1206*, the gene was deleted as described in Experimental procedures. PCR amplification confirmed that only a 1048-bp fragment was amplified from the Δ*HAH_1206* mutant instead of the 2083-bp fragment present in the parental *H. hispanica* strain. The deletion of *HAH_1206* was further confirmed by Southern blot. When genomic DNA was digested with *Ava* I and incubated with an 800-bp probe, a 2186-bp fragment was detected in the Δ*HAH_1206* mutant instead of a 3221-bp fragment seen in the parental strain, thus confirming *HAH_1206* deletion.

### HAH_1206 is not involved in N-glycosylation of the S-layer proteins

**Figure 2:**
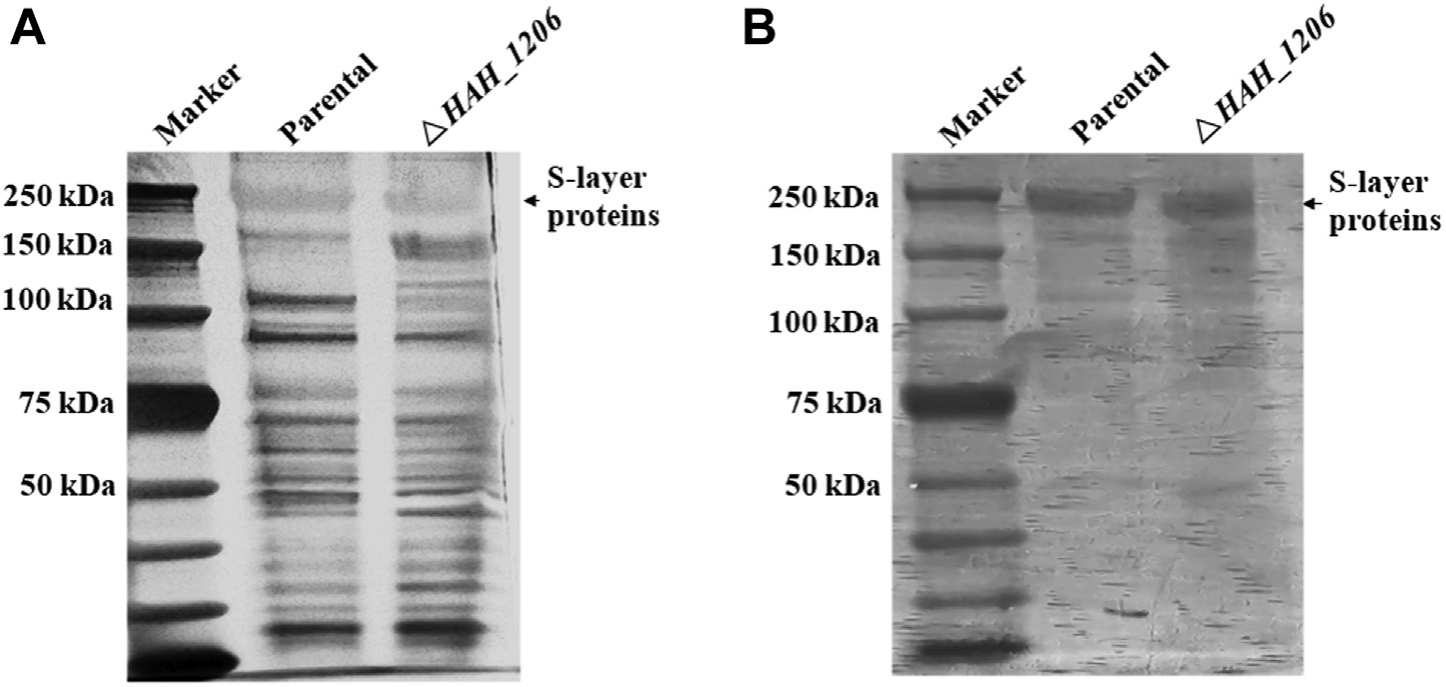
**Isolation and staining of S-layer glycoproteins.** Membrane proteins (36 μg) from the parental and mutant cells were separated by 7.5% SDS-PAGE and visualized by silver staining (*A*) and PAS glycoprotein staining (*B*). PAS, Periodic Acid-Schiff’s.

Previously, we showed that the two S-layer proteins of *H. hispanica* are decorated with an N-linked branched 6-sulfo-QuiNβ-(1,6)-[Glcα-(1,2)-]Gal trisaccharide and an O-linked Glc-1,4-Gal disaccharide (33). As *HAH_1206* lies within the gene cluster containing an *aglB* homolog encoding the archaeal oligosaccharyltransferase, we first asked whether glycosylation of the S-layer proteins was affected in the Δ*HAH_1206* strain. Accordingly, the S-layer proteins were extracted from *H. hispanica* parental or mutant cells cultivated in AS-168 medium at 37 °C, separated by SDS-PAGE, and subjected to silver and Periodic Acid-Schiff’s (PAS) staining. As shown in Figure 2, although silver staining revealed some proteins unique to either the parent or mutant strain or differing in amount between the two strains, the S-layer proteins from the Δ*HAH_1206* mutant were found at similar levels in both strains and migrated identically on the SDS-PAGE gel (Fig. 2*A*). It should be noted that SDS-PAGE does not separate the two S-layer proteins well (33). Furthermore, glycosylation of the S-layer proteins was not affected in the Δ*HAH_1206* mutant, as reflected by the similar PAS staining in the parental and mutant strains (Fig. 2*B*).

**Figure 3:**
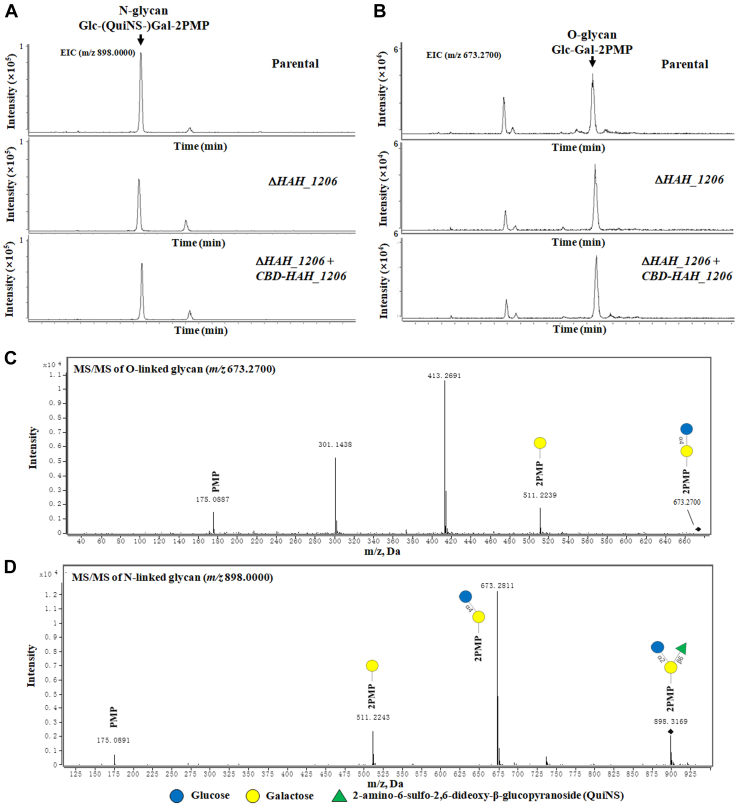
**Analysis of the glycans of the S-layer glycoproteins.** The [M + H]^+^ ions at *m/z* 673.2700 (*A*) and *m/z* 898.0000 (*B*) were extracted and identified in extracted ion chromatograms (EICs) and by LC-MS/MS (*C* and *D*).

In a second experiment, glycans were released from the S-layer glycoproteins by the One-Pot Release and Labeling (OPRAL) method (34). The released N- and O-glycans were separated by RP-HPLC using an Agilent HC-C18 column (250 × 4.6 mm, 5 μm) and analyzed by online RP-HPLC-UV-ESI-MS/MS. As shown in Figure 3, the glycans at *m/z* 898 and *m/z* 673 were identified as a di-1-phenyl-3-methyl-5-pyrazolone (PMP)–modified N-linked trisaccharide and an O-linked disaccharide by MS/MS, respectively. Both glycans also appeared in samples prepared from the Δ*HAH_1206* strain, indicating that HAH_1206 does not participate in N- or O-glycosylation of the *H. hispanica* S-layer glycoproteins.

### HAH_1206 contributes to the appearance of DolP-linked hexuronic acid and sulfated hexuronic acid

**Figure 4:**
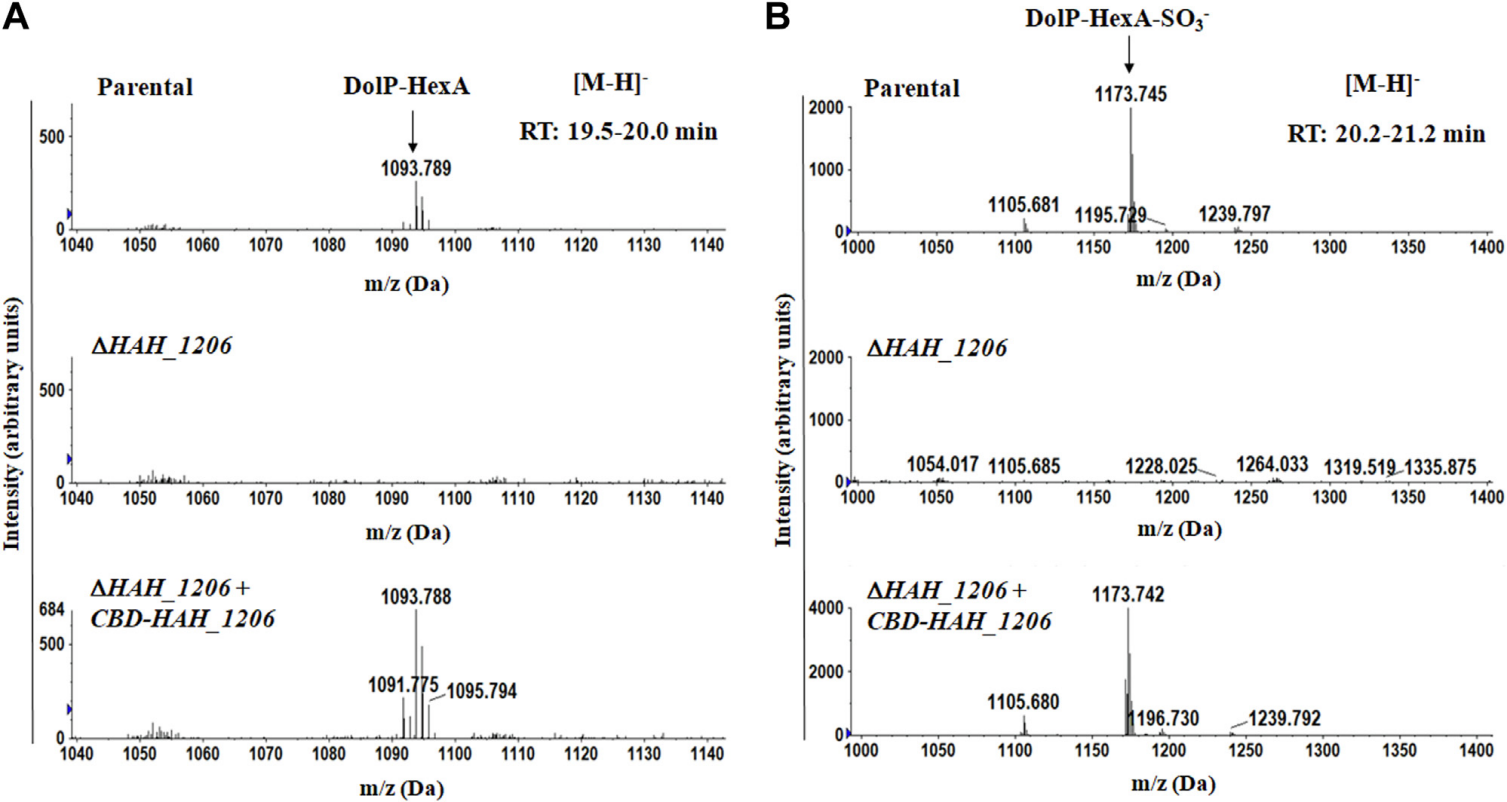
**Normal-phase LC-ESI MS analysis of C**_**60**_**DolP-linked sugars.** Total lipid extracts from *Haloarcula hispanica* parental, mutant, and complemented strains were analyzed by normal-phase LC-ESI MS as described in Experimental procedures. [M-H]^-^ ion peaks at *m/z* 1093.7 (*A*) and *m/z* 1173.7 (*B*) correspond to C_60_ DolP-linked HexA and HexA-SO_3_, respectively. Δ*HAH_1206*, mutant strain; Δ*HAH_1206*+*CBD-HAH_1206*, complemented strain. CBD, cellulose-binding domain; DolP, dolichol phosphate.

Since glycosylation of the *H. hispanica* S-layer glycoproteins was not affected in Δ*HAH_1206* cells (Fig. 3), it was not surprising to detect in the deletion strain DolP-hexose, DolP-dihexose, and DolP-QuiNS, all involved in S-layer protein glycosylation, in the deletion strain (Fig. S1). In contrast, analysis of the membrane fraction extracted from Δ*HAH_1206* cells by normal-phase LC-MS in the negative ion mode revealed that neither DolP-HexA nor DolP-HexA-SO_4_ (Fig. 4) was detected in the mutant, despite being present in the parent strain. This suggests HAH_1206 is involved in the appearance of these DolP-bound sugars in *H. hispanica*.

*H. hispanica* produces an acidic EPS consisting of the neutral sugars glucose (Glc), galactose (Gal), and mannose (Man) and an unidentified acidic component (21). EPSs extracted from the parental strain consists of GlcA, Man, Gal, and Glc at a 4:3.6:1.5:1 M ratio. However, analysis of the EPS from the Δ*HAH_1206* mutant showed only trace amounts of GlcA could be detected (35). To further assess whether HAH_1206 contributes to acidic EPS synthesis, DolP-linked sugars in the parental and mutant strains were extracted from the cell membrane, released with hydrofluoric acid, and analyzed by HPAEC-pulsed amperometric detection. These experiments detected GlcA in the membrane extract of the parental strain but not in the extract from the mutant (Fig. S2). Together, these results suggest that HAH_1206 is responsible for the synthesis of DolP-GlcA, the likely donor of acidic sugar residues in the acidic EPS of *H. hispanica*. Accordingly, *HAH_1206* was renamed *aepG*.

### *In vivo* and *in vitro* demonstration of AepG activity

**Figure 5:**
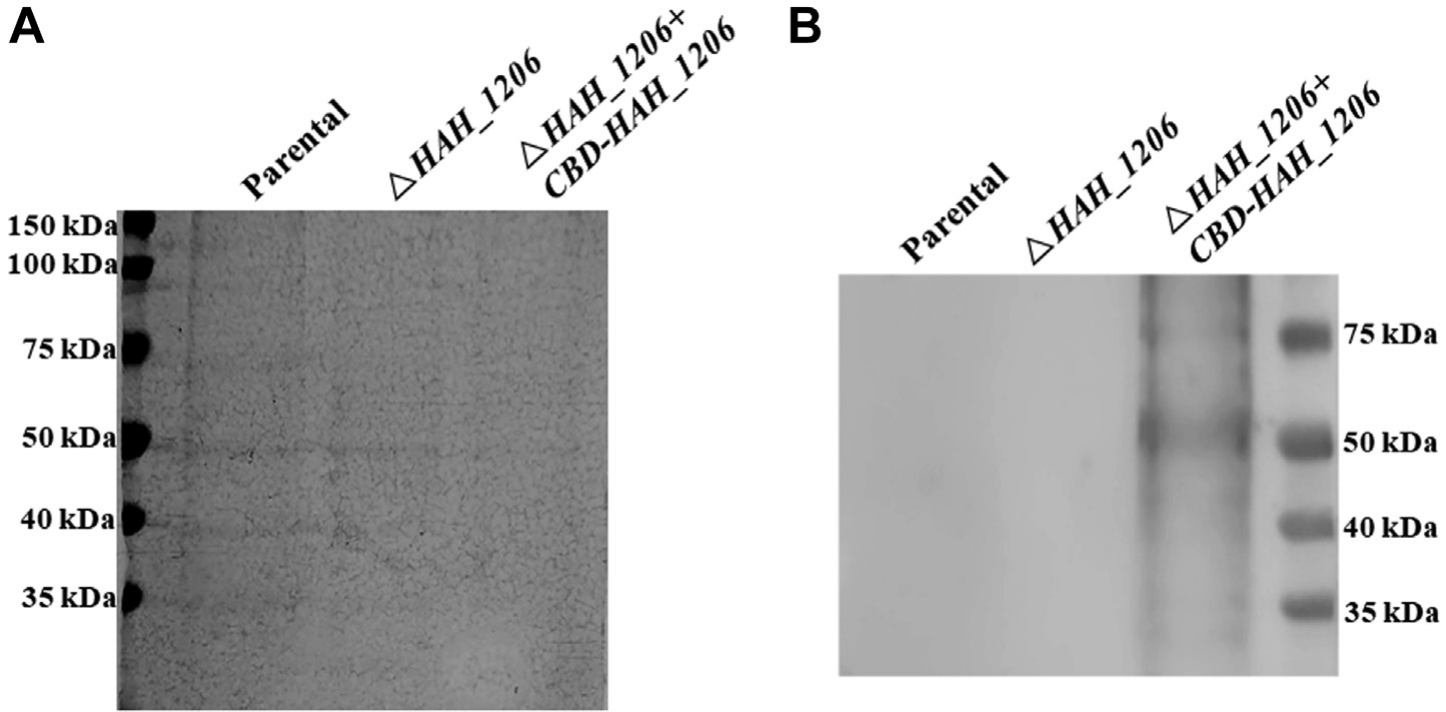
**Detection of CBD-tagged AepG in complemented strain.** Two milliliter aliquots of the cells were harvested, resuspended in 1 ml of solubilization buffer, and incubated at 37 °C for 2 h as described in Experimental procedures. After solubilization, CBD-tagged proteins were captured with cellulose. The cellulose pellets were collected, separated on 7.5% SDS-PAGE (*A*), and immunoblotted with antibodies against the cellulose-binding domain (*B*). aepG, archaeal exopolysaccharide glucuronosyltransferase; CBD, cellulose-binding domain.

To further address the role of AepG in DolP-GlcA synthesis, the deletion strain was complemented to express cellulose-binding domain (CBD)-tagged AepG (Fig. 5). Such complementation resulted in the appearance of both DolP-HexA and DolP-HexA(SO_3_) (Fig. 4).

**Figure 6:**
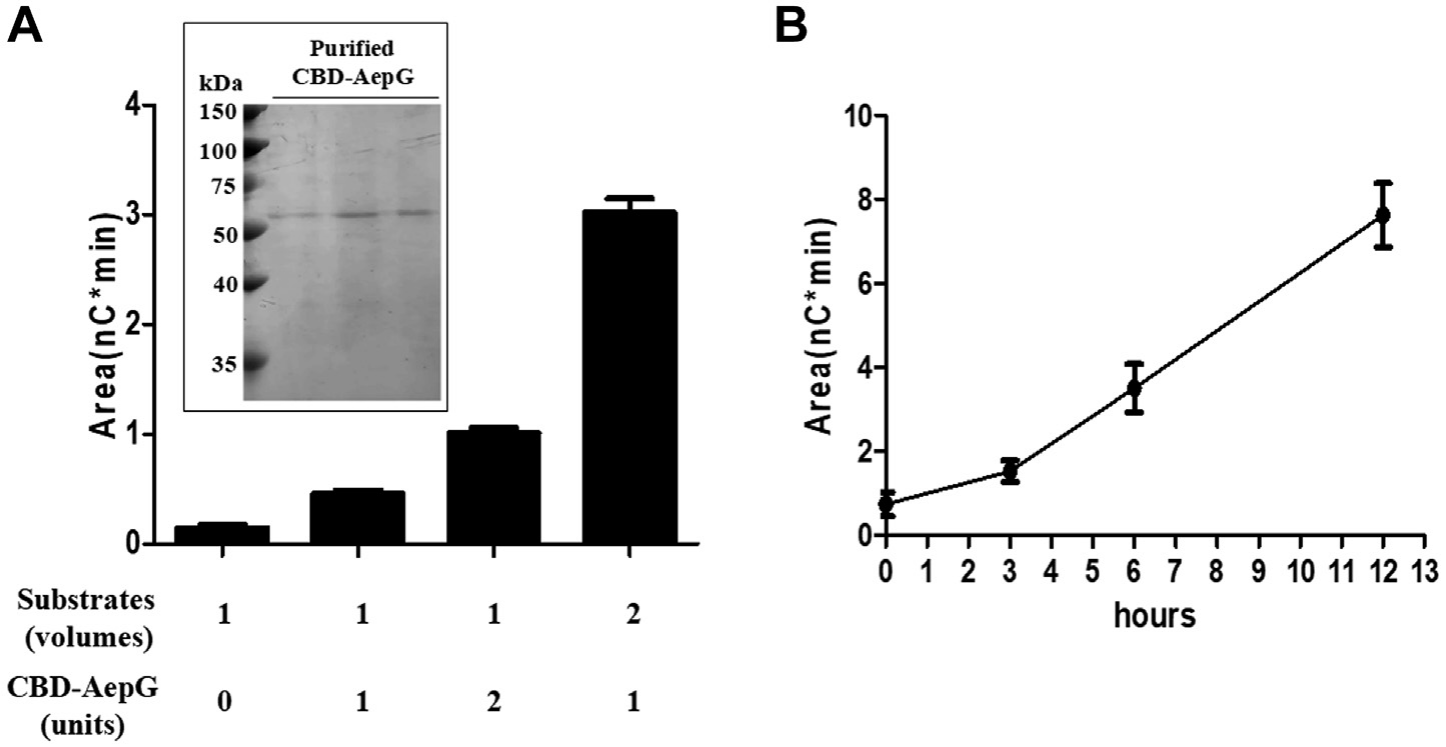
***In vitro* AepG activity assay.** AepG activity was assayed as described in Experimental procedures. In the assay, the reaction was initiated upon addition of 1 unit of CBD-tagged AepG into 1 volume of substrate in 5 ml of cell lysis buffer containing DNase I. After a reaction at 37 °C for 12 h, lipid-linked sugars were extracted by methanol and chloroform. Sugars were released from the lipids by HF and analyzed with HPAEC-PAD. *A*, effects of substrates or CBD-AepG on activity. Various amounts of substrates or purified CBD-AepG (insert) were added to the reaction mixture. After incubation at 37 °C for 12 h, the amount of DolP-linked GlcA was determined. *B*, effect of reaction time on activity. Three units of CBD-AepG were added into three volumes of substrates. After reactions at 37 °C for 3, 6, and 12 h, the amounts of DolP-linked GlcA were determined. aepG, archaeal exopolysaccharide glucuronosyltransferase; CBD, cellulose-binding domain; DolP, dolichol phosphate; GlcA, glucuronic acid; HF, hydrofluoric acid; PAD, pulsed amperometric detection.

The CBD-AepG expressed in the complemented strain was next used in an *in vitro* assay to test for glucuronosyltransferase activity. As C_60_-DolP is not commercially available, the cell pellet from the mutant containing this lipid was used as substrate. As shown in Figure 6, the addition of CBD-AepG to the reaction mixture led to both dose- and time-dependent increases in the amount of DolP-HexA. No DolP-HexA was generated when the assay was performed in the absence of CBD-AepG.

Together, these *in vivo* and *in vitro* results confirm that AepG is a DolP glucuronosyltransferase responsible for the synthesis of DolP-GlcA in *H. hispanica.*

### Phenotypes of the Δ*aepG* mutant

**Figure 7:**
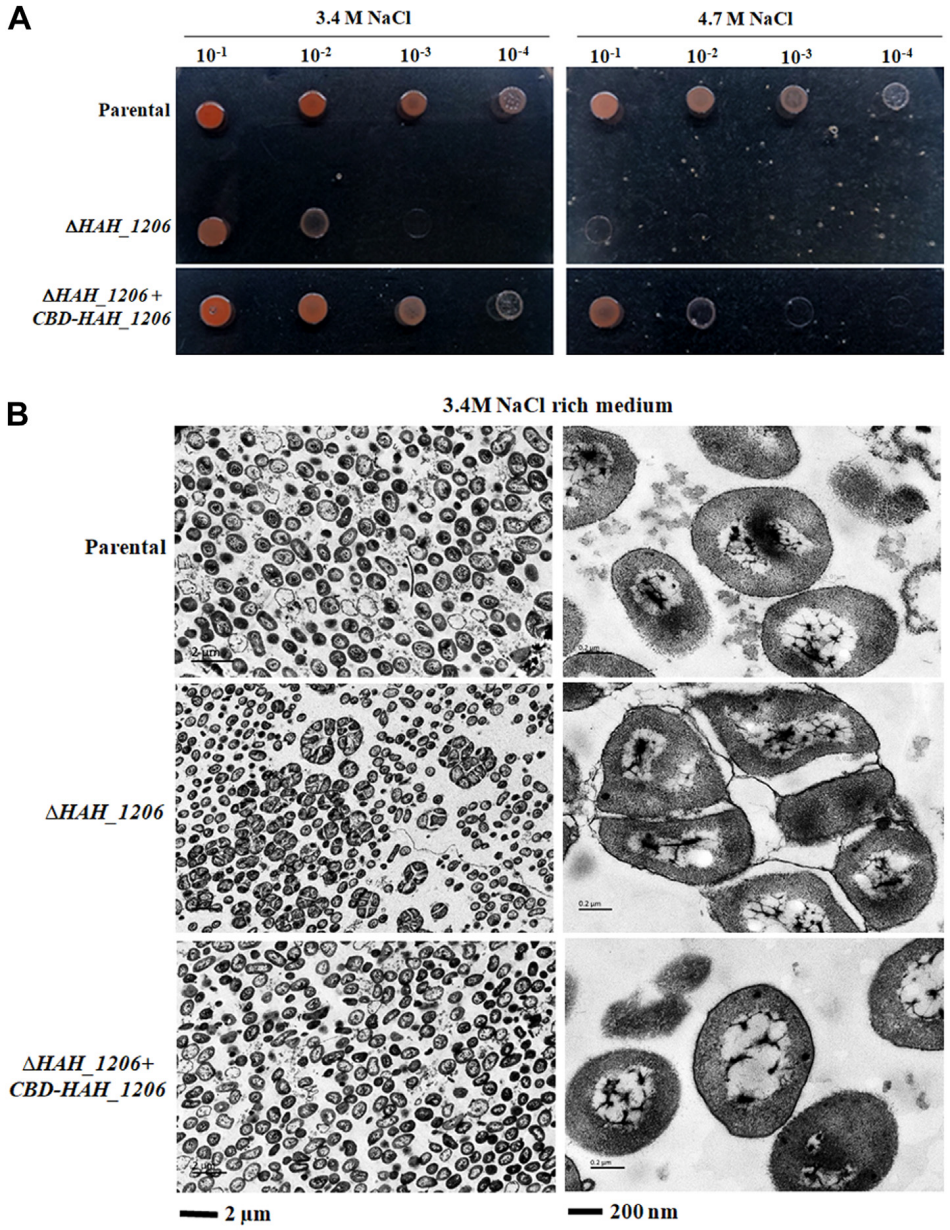
**Growth and morphology of the Δ*aepG* mutant strain.***A*, parental and mutant cells were spotted onto AS-168 agar plates containing different salt concentrations and incubated at 37 °C for 3 to 5 days. *B*, parental and mutant strain cells grown in medium containing 3.4 M NaCl were fixed, dehydrated, and embedded, as described in Experimental procedures. Sections were examined with an H-600 electron microscope (Hitachi). aepG, archaeal exopolysaccharide glucuronosyltransferase.

To evaluate the significance of the *aepG* gene, the mutant strain was grown under high salt conditions. On medium containing 3.4 M NaCl (normal growth conditions) or 4.7 M NaCl, the mutant showed significantly reduced growth (Fig. 7*A*). Transmission electron microscopy revealed the parental cells to be irregularly shaped and dispersed as single cells in the high salt medium. In contrast, Δ*aepG* cells formed aggregates (Fig. 7*B*). These observations suggest that AepG is required for proper growth in high salts. We showed that deletion of *aepG* resulted in the loss of DolP-HexA and DolP-HexA-SO_4_ (Fig. 5), the sugar donors for the negatively charged sugars of the acidic EPS. Indeed, only trace amounts of GlcA could be detected in the EPS from the Δ*HAH_1206* mutant (35). Therefore, it is reasonable to conclude that AepG is required for the synthesis of the negatively charged sugars of the acidic EPS, which is required for the separation and dispersal of *H. hispanica* cells in hypersaline surroundings.

## Discussion

Among microbial EPSs, bacterial and fungal EPSs have been most extensively studied (1, 4). Most bacterial and fungal EPSs are hetero-polysaccharides containing three or four different monosaccharides arranged in groups of 10 or less to form repeating units. Although some EPSs are neutral macromolecules, the majority are polyanionic due to the presence of uronic acids or ketal-linked pyruvate or inorganic residues. In bacteria, three pathways of EPS production have been reported, namely the Wzx/Wzy flippase-dependent pathway, the ABC transporter–dependent pathway, and the synthase-dependent pathway (9, 36, 37). None of the enzymes used to synthesize anionic uronic acid residues in EPSs have been identified to date.

In recent years, the study of extremophiles has gained increasing attention, not only from an evolutionary point of view but also for exploring the EPSs that allow these microorganisms to survive in extreme environments (19). Various haloarchaea produce EPSs (14, 15, 16, 17, 18, 19), yet little is known about archaeal EPS biosynthesis. *H. hispanica* can produce an acidic EPS consisting of GlcA, Man, Gal, and Glc at a 4:3.6:1.5:1 M ratio (21, 35), by a biosynthetic pathway that remains unknown.

The *H. hispanica HAH_1202-HAH_1214* gene cluster was predicted to encode N-glycosylation–related proteins based on homologies to components of the well-defined *H. volcanii* N-glycosylation pathway (22, 33). Specifically, *HAH_1214* was predicted to encode a homolog of *H. volcanii* AglJ that adds the first sugar of an N-linked pentasaccharide to a DolP carrier (32), *HAH_1202* is thought to encode the oligosaccharyltransferase AglB (38), and *HAH_1203* was predicted to encode a homolog of the *H. volcanii* hexuronic acid transferase AglG that adds GlcA to glucose-charged DolP (24). The *aepG* (*HAH_1206*) gene was predicted to encode a Group 2 family glycosyltransferase, specifically, a homolog of DolP mannose synthase (39). Based on these observations, we initially thought that *aepG* (*HAH_1206*) might be involved in *H. hispanica* protein glycosylation. However, analysis of glycans decorating the S-layer proteins of *H. hispanica* Δ*aepG* cells revealed that protein glycosylation was not affected in the mutant.

We have shown that GlcA residues were not detected in the EPS of the Δ*aepG* mutant (35), which also lacked DolP-linked GlcA. Using recombinant CBD-AepG and the cell lysate of the mutant, we determined AepG activity *in vitro*. These results, together with complementation studies, confirmed that AepG is a DolP glucuronyltransferase responsible for the synthesis of DolP-GlcA, the donor of acidic sugar residues in the *H. hispanica* EPS. Finally, we showed that deletion of *aepG* led to retarded growth and aggregation of *H. hispanica* cells. As AepG is responsible for the production of DolP-GlcA, presumably a donor of negatively charged sugars in the acidic EPS, it is reasonable to conclude that separation and dispersal of *H. hispanica* cells in a high salt environment involve the negatively charged GlcA and GlcA-SO_3_ residues of the EPS. Therefore, our results demonstrate that AepG is essential for the adaptation of *H. hispanica* to hypersaline surroundings. Additionally, we recently showed that the EPS from *H. hispanica* exhibited antiviral activity against SARS-CoV-2 but not the EPS from the Δ*aepG* mutant, suggesting an important role for the acidic sugar residues of the EPS in this antiviral activity (35). In summary, we report the first DolP glucuronyltransferase in archaea and also the first DolP-modifying glycosyltransferase contributing to EPS biosynthesis. Our results expand the diversity of pathways of EPS biosynthesis and may have a biotechnological potential for the biosynthesis of functional acidic polysaccharides.

## Experimental procedures

### Growth conditions

Strains and plasmids used in this study are listed in Table 1. *H.* *hispanica* DF60 (parental strain) and the Δ*aepG* mutant were cultured at 37 °C in AS-168 medium (5.0 g yeast extract, 5.0 g casein acids, 1.0 g Na-glutamate, 2.0 g KCl, 3.0 g Na_3_-citrate, 20.0 g MgSO_4_•7H_2_O, 200.0 g NaCl, 36.0 mg FeCl_2_•4H_2_O, 0.36 mg MnCl_2_•4H_2_O, brought to a final volume of 1000 ml with distilled water, pH 7.0–7.2.) (40).

**Table 1:** Strains and plasmids used in this study

Strain or plasmid	Description	Source or reference
Strains		
*H. hispanica* DF60	*H. hispanica* Δ*pyrF* (parental strain)	(40)
DF60in	*H. hispanica* Δ*pyrF* containing pHARΔ*HAH_1206* (pop-in)	This study
Δ*aepG*	*H. hispanica* Δ*pyrF*Δ*HAH_1206* (pop-out)	This study
Plasmids		
pHAR	4.0 kb; integration vector containing *pyrF* and its native promoter	(40)
pHARΔ*HAH_1206*	5.0 kb; pHAR containing DF60Δ*HAH_1206* for deletion of DF60 *HAH_1206*	This study
pWL-CBD-SecE	10kb; contains ampicillin and novobiocin resistance genes	(41)
pWL-CBD-HAH_1206	11 kb; expressing HAH_1206 protein	This study

### Construction of the *aepG* deletion mutant

The *aepG* deletion mutant was created by homologous recombination, as previously described (40). One 532-bp fragment containing the upstream flanking sequence of the *aepG* gene was amplified by PCR using the primers HAH_1206GLF and HAH_1206GLR (Table 2). A 516-bp DNA fragment containing the downstream flanking region of *aepG* was amplified by PCR using the primers HAH_1206GRF and HAH_1206GRR (Table 2). These two PCR products were sequenced and cloned into plasmid pHAR to generate plasmid pHARΔ*HAH_1206* (Table 1). The resulting plasmid was then transformed into the *H.* *hispanica* parental strain to delete the *aepG* gene by double-crossover homologous recombination.

**Table 2:** Oligonucleotides used in this study

Primer	Sequence (5′-3′)^a^
HAH_1206GLF	ATAAAGCTTATGATGTCGCCGAACTG
HAH_1206GLR	CAGGGTGTCAGCTATAGGTATGTGATGCCGTGACTGT
HAH_1206GRF	ACAGTCACGGCATCACATACCTATAGCTGACACCCTG
HAH_1206GRR	ATAAAGCTTGCACAGCCGAGGAGTA
HAH_1206REF	GGAATTCCATATGATGGCGACGACAGAGGACGTACG
HAH_1206RER	CGGGGTACCTCACTTGTTTTCCGCGCCACC
HAH_1206GCF	ATGGCGACGACAGAGGACGTAC
HAH_1206GCR	TCACTTGTTTTCCGCGCCAC

a Restriction endonuclease sites are underlined.

Deletion of *aepG* was confirmed by PCR using the flanking sequence primers HAH_1206GLF and HAH_1206GRR. Southern blot analysis of the mutant was performed using an 800-bp hybridization probe. Probe labeling and color detection with NBT/BCIP were performed using a DIG-High Prime DNA Labeling and Detection Starter Kit I (Roche).

### Complementation of the Δ*aepG* mutant

For complementation of the mutant, plasmid pWL-CBD-SecE containing an archaeal constitutive promoter PrP16 and the CBD from *Clostridium thermocellum* was employed (41). The *aepG* gene was amplified from genomic DNA using primers HAH_1206REF/HAH_1206RER (Table 2), in which *Nde* I and *Kpn* I restriction sites were introduced at the 5′- and 3′-ends, respectively. The *secE* gene in pWL-CBD-SecE was then replaced by the *aepG* gene to generate plasmid pWL-CBD-HAH_1206. Δ*aepG* cells were transformed with plasmid pWL-CBD-HAH_1206 using the PEG precipitation method (42). Transformants were confirmed by PCR analysis using primers HAH_1206GCF/HAH_1206GCR (Table 2) and DNA sequencing. Expression of CBD-tagged AepG was confirmed by Western blot.

### Western blot

Capture and immunoblot of CBD-tagged protein were performed as described previously with slight modifications (42, 43). Briefly, 2 ml aliquots of the cells were harvested (3000 rpm in a microfuge for 10 min). The cells were resuspended in 1 ml of solubilization buffer (1% Triton X-100, 1.8 M NaCl, 50 mM Tris–HCl, pH 7.2) containing 10 μg/ml DNase I and incubated at 37 °C for 2 h. After solubilization, 50 μl of 10% (w/v) cellulose were added and the mixture was incubated at room temperature for 60 min. Then, the cellulose pellets were collected by centrifugation at 12,000 rpm for 10 min, washed twice with washing buffer (2 M NaCl, 50 mM Tris–HCl, pH 7.2), separated on 7.5% SDS-PAGE, and transferred to a nitrocellulose membrane. The CBD-tagged proteins were detected with polyclonal rabbit anti-CBD antibodies (1:2000 dilution). Alkaline phosphatase–conjugated anti-rabbit antibodies (Bio-Rad) were used as secondary antibodies (1:5000 dilution). Antibody binding was detected using BCIP/NBT Western blotting detection reagent (Pierce).

### Isolation of S-layer proteins

*H. hispanica* cells cultivated in 100 ml of AS-168 medium at 37 °C were collected and resuspended in a basal salt solution containing the same ionic composition as AS-168 medium. After sonication, the supernatant was collected by centrifugation at 7000*g* for 20 min and further ultracentrifuged at 250,000*g* at 4 °C for 1 h. The pelleted membrane proteins were dissolved in deionized water and quantified by Bradford assay (44). Membrane proteins (36 μg) from the parental or mutant were separated by 7.5% SDS-PAGE and visualized by silver staining (Sangon Biotech) or PAS reagent (Thermo Fisher Scientific).

### Release and labeling of glycans from the S-layer proteins

*H. hispanica* cells cultivated in 100 ml of AS-168 medium at 37 °C were collected by centrifugation at 7000*g* for 20 min and resuspended in 10 ml washing buffer. The S-layer proteins were extracted by the addition of 1 ml 0.5 M EDTA (pH 8.0). After incubation at 37 °C for 3 h, the mixture was centrifuged at 7000*g* for 20 min. The supernatant was collected, precipitated with 15% trichloroacetic acid on ice for 30 min or 4 °C overnight. The precipitated S-layer proteins were collected by centrifugation, washed with ice-cold acetone, and air-dried for further use.

Glycans on the S-layer proteins were released and labeled by the OPRAL method with slight modifications (34). Briefly, 0.35 g PMP were dissolved in 0.5 ml of a 1:1 (v/v) mixture of methanol and aqueous ammonium hydroxide (26%-28%, v/v) in a 1.5 ml centrifuge tube by heating and shaking in a water bath at 80 °C. Once the PMP had dissolved, a 1:1 (v/v) mixture of methanol and aqueous ammonium hydroxide was added to bring the final volume to 1 ml, followed by rapid mixing with a multivortex mixer. Subsequently, lyophilized S-layer proteins were dissolved in 1 ml of the reaction solution in a 1.5 ml screw-cap tube, followed by incubation at 80 °C for 16 h. When the reactions were finished, the reaction mixture was neutralized by adding glacial acetic acid drop-wise with shaking.

Glycans obtained using the OPRAL method were transferred into a new centrifuge tube, and a three-fold volume of dichloromethane was added, followed by violent shaking and centrifugation at 12,000×rpm for 10 min. This step was repeated until no intermediate layer appeared. The aqueous layer was transferred into another 2 ml centrifuge tube and concentrated to complete dryness using a SpeedVac concentrator. Finally, the dried glycans were redissolved in 1 ml of deionized water and loaded onto a Sep-Pak C18 cartridge prewashed with 25 ml of 25% acetonitrile (ACN) containing 0.1% TFA and then 5 ml 0.1% TFA. When the sample was fully absorbed, the column was washed with 3 to 5 ml 0.1% TFA to remove hydrophilic impurities, and the target glycans were eluted using 1 ml of 25% aqueous ACN containing 0.1% TFA solution. The elution fraction was concentrated and stored at −20 °C for further use.

### Glycan analysis by HPLC-MS/MS

The PMP-labeled glycans were dissolved in ddH_2_O. HPLC-MS/MS analysis was conducted on an Agilent 1290 HPLC System using an Agilent HC-C18 column (250 × 4.6 mm, 5 μm) with a flow rate of 1.0 ml/min. MS data were collected in the auto-MS/MS mode. The solvent system used was a linear gradient of 25% to 29% solvent B (ACN) in solvent A (10 mM aqueous ammonium acetate, titrated to pH 5.5 with glacial acetic acid) over a period of 20 min. The column temperature was set at 40 °C. The sample injection volume was 100 μl. The column was cleaned using 100% ACN for 5 min. Data acquisition was performed using Agilent MassHunter Workstation Software-Qualitative Analysis.

### Analysis of total lipid extracts

Total lipid extracts from *H. hispanica* parental, Δ*aepG* mutant, and complemented strains were analyzed by normal-phase LC-ESI MS as described previously (45). MS spectra of the major species were obtained in the negative ion mode.

### *In vitro* assay of AepG activity

The mutant cells were grown in 100 ml AS-168 medium and harvested by centrifugation when the A_600_ reached 1.0. The cells were resuspended in 5 ml of cell lysis buffer (5 mM MgCl_2_, 0.13% NP-40, 50 mM Tris–HCl, pH 8.0) containing DNase I and incubated at 37 °C with shaking at 200 rpm for 2 h to release the cell lysate, which was considered as one volume of substrates for use in assaying AepG activity.

The complemented strain was grown in 100 ml AS-168 medium to an A_600_ of 1.0. The cells were harvested by centrifugation, resuspended in 10 ml of solubilization buffer (1% Triton X-100, 1.8 M NaCl, 50 mM Tris–HCl, pH 7.2,), and incubated at room temperature for 2 h, followed by the addition of 0.15 g cellulose. After 60 min incubation at room temperature, the cellulose pellets were collected by centrifugation at 12,000 rpm for 10 min, washed twice with washing buffer (2 M NaCl, 50 mM Tris–HCl, pH 7.2), and defined as 1 unit of CBD-AepG for use in assaying AepG activity.

The assay was initiated by adding 1 unit of CBD-AepG into 1 volume of substrates (in 5 ml cell lysis buffer containing DNase I). After a reaction at 37 °C for 12 h, the mixture was centrifuged (8000*g* for 10 min). Total lipids in the supernatant were extracted with H_2_O:MeOH:CH_3_Cl (3.8:1:1), air-dried, and then hydrolyzed with 250 μl hydrogen fluoride on ice for 3 h. The released lipid-linked monosaccharides or glycans were dried with a hair dryer and dissolved in H_2_O:MeOH:CH_3_Cl (3.8:1:1). The water phase was collected, vacuum-dried, dissolved in water, and subjected to HPAEC-PDA analysis.

### Analysis of monosaccharides by HPAEC-pulsed amperometric detection

Standard and samples were separated by HPAEC and detected by pulsed amperometric detection (Dionex) using a CarboPack PA10 (2 × 250 mm) column (LCPackings). Acidic GlcA and GalA were eluted by 10 mM NaOH and 10 mM CH_3_COONH_4_ with 1 ml/min flow rate at a retention time of 25 min.

### Reverse transcription PCR

Total RNA was isolated using TRIzol Reagent (LIFE). Complementary DNA synthesis was performed with Fast Quant RT Kit (TIANGEN). Reverse transcription PCR was performed as described previously (22). Quantitative RT-PCR of *HAH_1206* was performed using SYBR Premix Ex Taq (Takara) with a primer pair of 5′-GGCCAGATATCCACGATTTC-3′ and 5′-3′GATGGTTAACTGGGATTTCTG. The 16S RNA gene was used as an internal control using a primer pair of 5′-TTGCGGGCTGAAACCCG-3′ and 5′-TCCTCTACGGCTACCTTGTTAC-3′.

### Transmission electron microscopy

*H. hispanica* parental and mutant cells in 3.4 M NaCl-containing buffer were fixed in 2.5% glutaraldehyde in 0.1 M phosphate buffer, pH 7.0, for 4 h or overnight at 4 °C, washed three times in 0.1 M phosphate, postfixed in 1% osmium tetroxide in 0.1 M phosphate for 2 to 4 h, incubated in 30%, 50%, 70%, 85%, 95%, and 100% methanol for 15 to 20 min at each concentration, and post-fixed in 2% uranyl acetate-30% methanol. The cells were rinsed, dehydrated, and embedded in Epson 812 for the floating sheet method. Sections were examined with an H-600 electron microscope (Hitachi).

## Data availability

The data that support the findings of this study are available from the corresponding author upon reasonable request.

## Supporting information

This article contains supporting information.

## Conflict of interest

The authors declare they have no conflicts of interest with the contents of this article.
