# Supplementary material for: AepG is a glucuronosyltransferase involved in acidic exopolysaccharide synthesis and contributes to environmental adaptation of Haloarcula hispanica
Source: J Biol Chem. 2023 Jan 13;299(2):102911. doi: 10.1016/j.jbc.2023.102911 (PMC9943897; doi:10.1016/j.jbc.2023.102911)
Supplement: Supplementary figures [file mmc1.pdf]

Supplementary figures

AepG is a glucuronosyltransferase involved in acidic exopolysaccharide synthesis and contributes to environmental adaptation of *Haloarcula hispanica*

Caixia Pei[1,2,#], Hua Lu[1,3,#], Jiayin Ma[4], Jerry Eichler[5], Ziqiang Guan[6], Linlu Gao[1,2], Li Liu[4], Hui Zhou[1], Jinghua Yang[1,2,*], Cheng Jin[1,2,*]

[1] State Key Laboratory of Mycology, Institute of Microbiology, Chinese Academy of Sciences, Beijing 100101, China; [2] University of Chinese Academy of Sciences, Beijing 100049, China; [3] Department of Pharmacology, UT Southwestern Medical Center, 6001 Forest Park Rd., Dallas, TX 75390, USA; [4] Glycomics and Glycan Bioengineering Research Center (GGBRC), College of Food Science and Technology, Nanjing Agricultural University, Nanjing 210095, China; [5] Department of Life Sciences, Ben Gurion University of the Negev, Beersheva 84105, Israel; [6] Department of Biochemistry, Duke University Medical Center, Durham NC 27710, USA.

[#] These authors contributed equally to this work.

*Corresponding authors:

Prof. Cheng Jin (jinc@im.ac.cn) or Dr. Jinghua Yang (yangjh@im.ac.cn)

Running Title:

AepG is a dolichol phosphate glucuronosyltransferase

Keywords: Archaea, carbohydrate biosynthesis, dolichol phosphate, enzyme, glucuronic acid, glycosyltransferase, *Haloarcula hispanica*

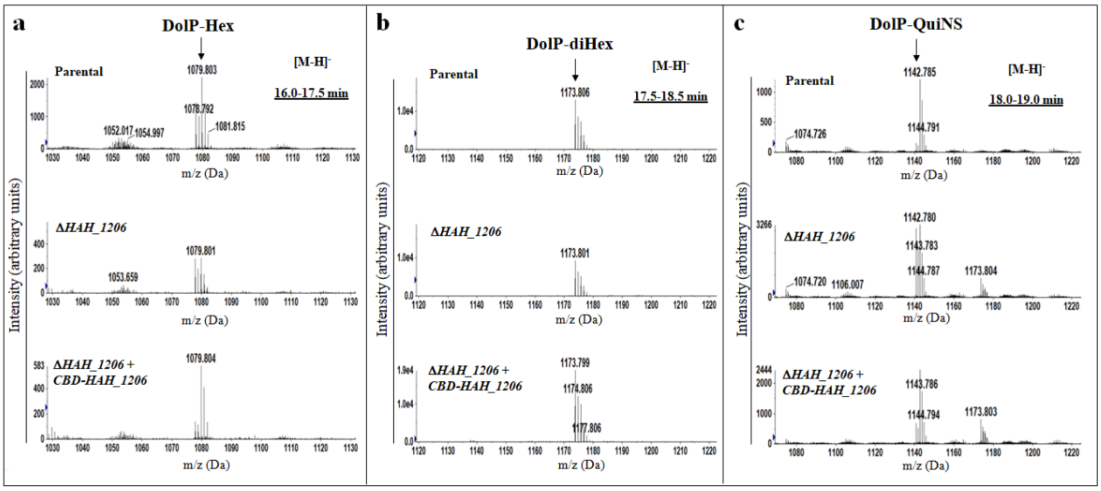

Supplementary Fig. 1 Normal-phase LC-ESI MS analysis of C_{60} DolP-linked sugars. The mass spectra averaged from spectra acquired from the parental, mutant (Δ*HAH_1206*) and complemented (Δ*HAH_1206*+*CBD-HAH_1206*) strains during the 16.0-17.5 min, 17.5-18.5 min and 18.0-19.0 min windows, respectively. $[M\text{-}H]^-$ ion peaks at *m/z* 1079.8 (**a**), *m/z* 1173.8 (**b**) and *m/z* 1142.7 (**c**) correspond to C_{60} DolP-Hex, DolP-diHex and DolP-QuiNS, respectively.

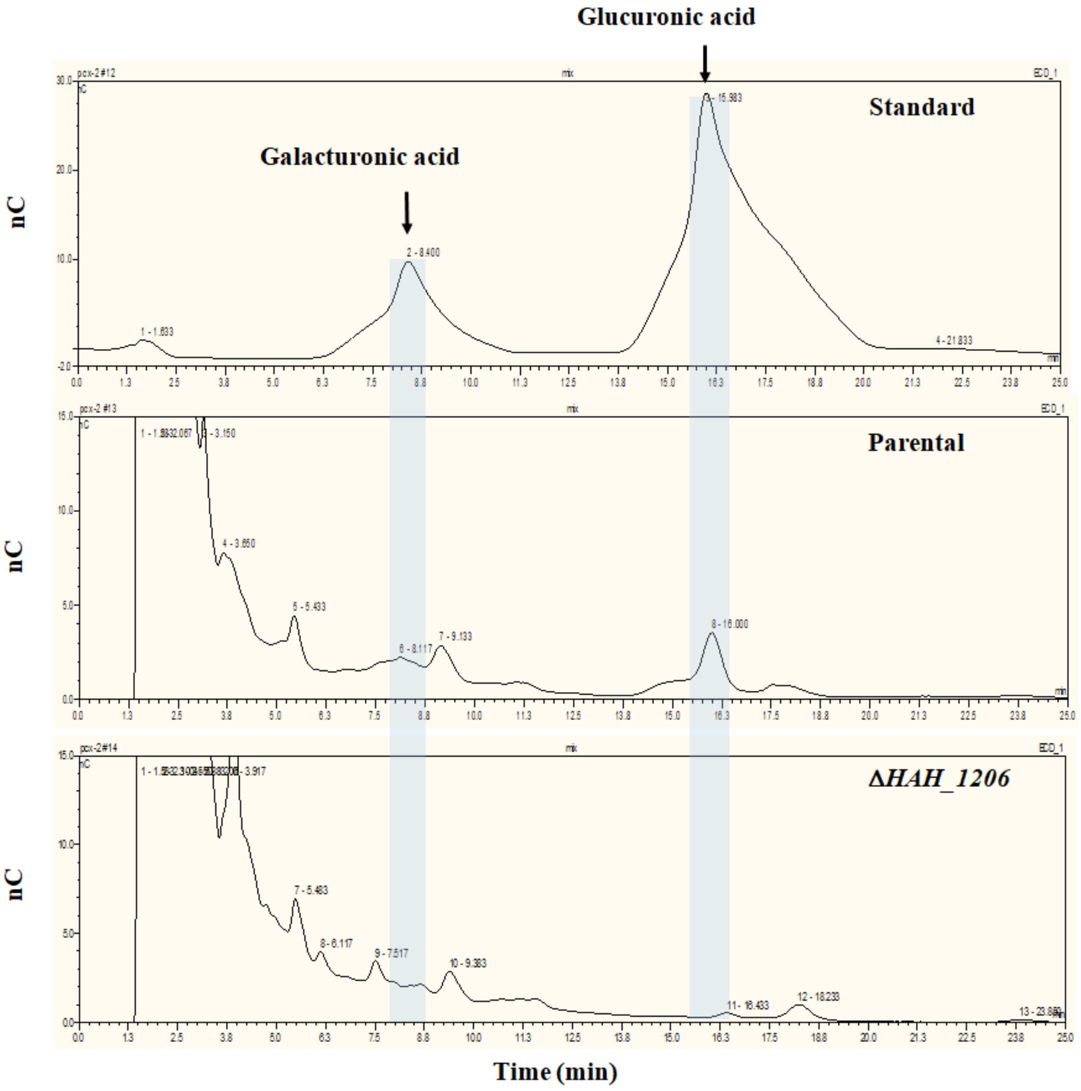

Supplementary Fig. 2 Analysis of DolP-linked sugars in the parental and mutant strains. DolP-linked acidic sugars were extracted from the cell membrane, released with hydrofluoric acid, and analyzed by HPAEC-PAD (Dionex) as described in Experimental procedures.
